# Neurophobia in health profession students and practitioners: a systematic review and synthesis without meta-analysis (SWiM)

**DOI:** 10.1007/s10072-026-09092-3

**Published:** 2026-06-09

**Authors:** Sara Garces-Arilla, Silvia Collado, Vanesa Hidalgo, Magdalena Mendez-Lopez, Camino Fidalgo

**Affiliations:** 1 Departamento de Psicología y Sociología, Facultad de Ciencias Sociales y Humanas, Universidad de Zaragoza, 44003, Teruel, Spain; 2 Laboratorio de Neurociencia Social Cognitiva, Universidad de Valencia, Valencia, 46016 Spain; 3 Instituto de Investigación Sanitaria Aragón (IIS), Aragón, Zaragoza, Spain

**Keywords:** Neurophobia, Health professions education, Measurement instruments, Prevalence, Educational interventions

## Abstract

**Background:**

Neurophobia, defined as the fear of or aversion to neurology and neuroscience, is a challenge across health sciences and may contribute to workforce shortages. Previous reviews have exclusively focused on medical populations and have not systematically addressed measurement variability or the effects of educational interventions.

**Objective:**

This systematic review builds on previous studies by analyzing neurophobia across the health sciences, focusing on prevalence rates, measurement instruments, associated factors, and the effects of educational interventions.

**Methods:**

A systematic search was conducted following PRISMA guidelines. Eligible studies explicitly measured neurophobia and provided details of the measurement tool used. A structured narrative synthesis was performed following synthesis without meta-analysis (SWiM) principles and methodological quality was assessed using the Mixed Methods Appraisal Tool.

**Results:**

21 studies fulfilled the inclusion criteria. These were conducted across diverse countries, and predominantly involved medical students and physicians in training, with dental and veterinary students also represented. Measurement instruments showed substantial heterogeneity and were classified into four domains: affective response, cognitive appraisal, capability beliefs, and motivational consequences. Prevalence rates ranged from 19% to 66%. Higher interest and greater clinical exposure were associated with lower neurophobia, whereas gender and academic progression showed inconsistent associations. Evidence on educational interventions was limited and heterogeneous, although clinical or applied approaches suggested reductions in neurophobia.

**Conclusion:**

Overall, findings highlight variability in prevalence and measurement approaches. Associations with clinical exposure and reductions following clinically or applied educational interventions suggest that neurophobia might be less strongly associated with neurosciences’ intrinsic complexity and more strongly associated with modifiable educational factors.

## Introduction

Neurophobia, the fear of or aversion to neuroscience and clinical neurology, is a significant barrier in health education. First described by Jozefowicz [1], it is often attributed to the difficulty of applying complex theoretical knowledge in neuroscience to clinical practice. This phenomenon has been widely documented in medical training [2, 3], and medical students consider neurology one of the most challenging disciplines due to the complexity of neuroanatomy and the inherent difficulty of neurological examination [3]. Medical students and health practitioners may be less likely to choose neurology and related fields as a professional specialization due to their perceived difficulty [4]. Also, health practitioners have reported lower confidence in the assessment of patients with neurological disorders compared to patients without these disorders [5], suggesting that neurophobia may have important implications for healthcare delivery. In this context, neurophobia may be associated with reduced recruitment into neurology, which is particularly concerning given the increasing incidence and prevalence of neurological diseases [6, 7].

Neurophobia has been primarily described among medical students [2, 8], but recent studies also report its presence in other health-related disciplines, such as dental [9] and veterinary students [10]. For instance Javaid et al. [11] examined a cross-disciplinary cohort of medical, dental, occupational therapy, and speech and language students, and found that neuroanatomy was consistently perceived as one of the most difficult subjects across all groups. Similarly, Mendez-Lopez et al. [12] found that psychology students experience significant difficulties learning neuroanatomy, particularly due to challenges with spatial visualization and understanding relationships among brain structures. Together, these findings suggest that the difficulties underlying neurophobia are not exclusive to medical training, but may reflect more general challenges associated with the teaching and learning of complex neuroscientific content. This effect may extend beyond undergraduate education, as studies with residents show persistent deficits in competencies, clinical exposure, and advanced training [13], suggesting structural limitations in neuroscience education across the entire training continuum.

Given the difficulties students experience when learning neurology or neurosciences, fields in which neuroanatomy is a core component of the curriculum, several studies have focused on facilitating neuroanatomy learning for medical students as well as for those from other health-related disciplines (see [14] for a review). These studies have evaluated the effectiveness of different teaching methodologies by measuring student performance in neuroanatomy texts and perceived satisfaction with the subject [12, 15, 16]. Systematic reviews highlight the use of innovative tools, such as 3D models, augmented reality, and flipped classrooms, as promising strategies for enhancing the study of neuroscience [14, 17]. However, only a few recent studies have focused explicitly on examining the potential benefits of interventions aimed at reducing neurophobia [18–22], and their results have not been systematically synthesized.

Additionally, the available literature reflects a lack of standardization in the conceptualization and assessment of neurophobia, which hinders the ability to compare findings across studies and to determine its prevalence and influencing factors. Regarding measurement models, approaches vary widely. Specifically, some studies use single-item questions, often capturing only affective responses such as fear or anxiety [22]. Other studies use multi-item scales that combine cognitive appraisal (e.g., difficulty, complexity, knowledge burden) and capability beliefs (e.g., confidence, self-efficacy) [9, 21]. More recently, however, researchers have broadened this operationalization by developing measurement instruments that explicitly integrate motivational consequences alongside the established cognitive and capability domains [10, 20].

Previous systematic reviews of neurophobia, while offering valuable insights, have not fully addressed these emerging complexities. Specifically, they have primarily focused on medical populations [23–25], thereby overlooking evidence that neurophobia also taxes students and practitioners across broader health-related disciplines. In addition, they have not systematically addressed the considerable variability in how the construct is defined and measured [23–25]. Furthermore, although various educational strategies have been proposed to reduce neurophobia, these systematic reviews either omit [23–25] or fail to systematically synthesize interventions specifically targeting this aversion [24]. To address these limitations, we conducted the present systematic review aiming to: (a) broaden the scope of analysis to include multiple health science disciplines; (b) systematically identify and classify the instruments used to measure neurophobia according to their underlying conceptual domains; (c) examine the variability in reported prevalence in relation to these measurement approaches; (d) synthesize factors associated with the development of neurophobia; and (e) synthesize the evidence for educational interventions designed to dampen it. By doing so, we aim to clarify the current state of knowledge and provide a clear roadmap for future research.

## Materials and methods

The systematic review was conducted in accordance with the Preferred Reporting Items for Systematic Reviews and Meta-analyses (PRISMA) guidelines [26], and our protocol was registered with PROSPERO (CRD420251056085) in May 2025. Given the substantial heterogeneity in study designs, participant populations, neurophobia measures, and reported outcomes, conducting a meta-analysis was deemed inappropriate; therefore, we undertook a structured narrative synthesis following the SWiM (Synthesis Without Meta-analysis) principles [27].

### Search strategy

Systematic searches were conducted in various databases: PubMed, Scopus, and Web of Science. The following combination of search strings was used to identify relevant studies: “neurophobia” OR “neurophobic” OR “fear of neurology” OR “fear of neuroscience” OR “neurology anxiety” OR “anxiety about neurology” OR “anxiety in neurology” OR “aversion to neurology” OR “aversion to neuroscience” OR “perceived difficulty of neurology” OR “difficulty learning neurology” OR “neurology is difficult” OR “confidence in neurology” OR “lack of confidence in neurology” OR “self-efficacy in neurology.” The study selection process ended on March 20, 2026.

### Inclusion and exclusion criteria

The results were manually screened according to the authors’ predefined inclusion and exclusion criteria. Inclusion criteria were as follows: (1) empirical quantitative, qualitative, or mixed-methods studies, (2) studies must explicitly measure neurophobia and provide a detailed description of the method used for its assessment; (3) intervention studies must measure neurophobia; (4) studies must involve students or professionals with a relevant connection to neuroscience, neuroanatomy, or neurology. Exclusion criteria were as follows: (1) abstracts, conference papers, literature reviews, academic dissertations, books, book sections, theses, as well as letters, opinion pieces, commentaries, and points of view; (2) studies not written in English; (3) studies not published in peer-reviewed academic journals; (4) studies that did not measure neurophobia or did not report how it was measured.

### Selection of papers

Two independent reviewers performed the study selection in two screening stages. First, titles and abstracts were reviewed. Second, full texts were assessed for inclusion. Any conflicts between reviewers were resolved through discussion, and a third reviewer was consulted if consensus could not be reached.

### Data extraction and narrative review synthesis strategy

**Fig. 1:**
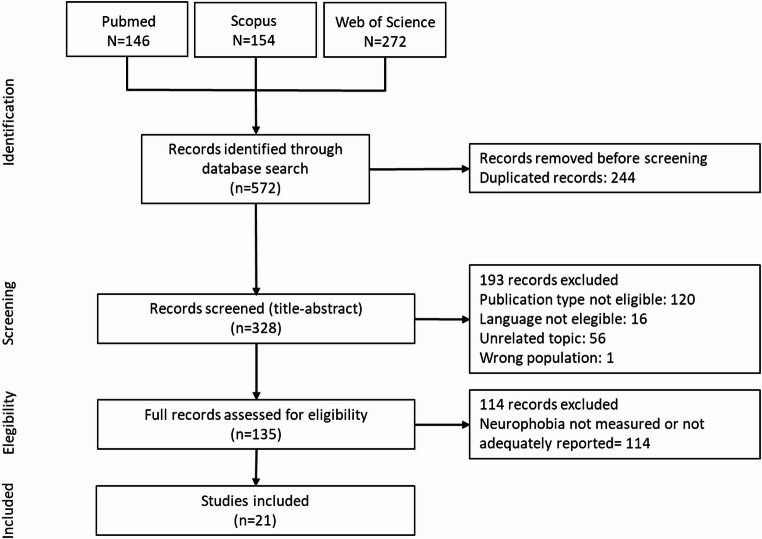
Systematic review flowchart

Initially, a total of 572 records were identified through database searching (PubMed: 146; Scopus: 154; Web of Science: 272). Following the removal of duplicates (*n* = 244), the remaining 328 records were screened based on title and abstract. Of these, 193 records were excluded (publication type not eligible: 120; language not eligible: 16; non-related topic: 56; wrong population: 1). Next, we assessed the remaining 135 full-text articles for eligibility. During this phase, 114 were excluded because neurophobia was not explicitly measured or the data were not adequately reported. Ultimately, 21 articles fulfilled all criteria and were selected for inclusion in this review (Fig. 1).

First, we extracted data from individual studies to describe key findings related to neurophobia prevalence, measurement tools, associated factors, and educational interventions. Because of the substantial heterogeneity in study designs, participant populations, neurophobia measures, and reported outcomes, a meta-analysis was deemed inappropriate. To improve comparability, we classified the neurophobia measurement instruments into four conceptual domains according to the specific psychological constructs they assessed: affective response (i.e., fear, aversion, anxiety), cognitive appraisal (i.e., perceived difficulty or complexity), capability beliefs (i.e., self-confidence, perceived knowledge, self-efficacy), and motivational consequences (i.e., interest). Specifically, when multidimensional instruments were used, we identified domains included within each instrument and mapped them to these categories (Table 1). Accordingly, we conducted a structured narrative synthesis following the SWiM principles.

**Table 1:** Measurement instruments and operational definitions of neurophobia across included studies

Author (Year)	Population	Instrument	Neurophobia items (*n*)	Response format	Operational definition of neurophobia	Domains assessed	Psychometric properties
Aka et al. (2025) [18]	Medical students	Neuro-Q [21]	5	5-point Likert scale	Neuro-Q score > 16	Cognitive appraisal and capability beliefs	Validated instrument (as stated by authors; not detailed)
Aka et al. (2025) [34]	Medical students and interns	Schon questionnaire [3]	2	5-point Likert scale	Combined confidence and difficulty score ≤ 4	Cognitive appraisal and capability beliefs	Not reported
Ali et al. (2025) [35]	Medical students	Self-developed questionnaire	2	5-point Likert scale	Combined confidence and difficulty score ≤ 4	Cognitive appraisal and capability beliefs	Not reported
Ali et al. (2026) [38]	Interns/primary care and residents	Self-developed questionnaire	2	5-point Likert scale	Combined confidence and difficulty score ≤ 4	Cognitive appraisal and capability beliefs	Not reported
Chua et al. (2020) [30]	Family medicine specialist trainees	Self-developed questionnaire adapted from McCarron et al. [46] and Kam et al. [39]	1	5-point Likert scale	Score ≥ 4 on perceived difficulty in neurology	Cognitive appraisal	Not reported
Corigliano et al. (2024) [9]	Dental students	Neuro-Q [21]	5	5-point Likert scale	Neuro-Q score ≥ 16	Cognitive appraisal and capability beliefs	Internal consistency low: Cronbach’s alpha = 0.53
Fantaneau et al. (2014) [2]	Medical students	Self-developed questionnaire	2	4-point Likert scale	Agreement with one or both fear-related items (afraid of clinical neurology and academic neuroscience)	Affective response	Not reported
Farrag et al. (2025) [36]	Medical students	Neuro-Q [21]	5	5-point Likert scale	Neuro-Q score > 16 = neurophobia; Neuro-Q score > 18 = marked neurophobia	Cognitive appraisal and capability beliefs	Validated instrument (as stated by authors; not detailed)
Han et al. (2023) [32]	Medical students and resident trainees	Self-developed questionnaire	2	5-point Likert scale	Combined confidence and difficulty score ≤ 4	Cognitive appraisal and capability beliefs	Not reported
Holroyd et al. (2025) [19]	Medical students	Self-developed questionnaire	2	5-point Likert scale	Agreement with two fear-related items (afraid of neurology and neuroscience)	Affective response	Not reported
Jukna et al. (2023) [8]	Medical students	Self-developed questionnaire based on Schon et al. [3]	2	5-point Likert scale	Combined confidence and difficulty score ≤ 4	Cognitive appraisal and capability beliefs	Not reported
Kam et al. (2013) [39]	Medical students and junior doctors	Self-developed questionnaire based on Schon et al. [3]	2	5-point Likert scale	Combined confidence and difficulty score ≤ 4	Cognitive appraisal and capability beliefs	Not reported
Lambea-Gil et al. (2023) [31]	Medical students	Self-developed questionnaire	1	5-point Likert scale	High or very high fear or rejection of neurology (Likert 4–5)	Affective response	Not reported
McElligott et al. (2025) [20]	Medical students	NCM	9	5-point Likert scale	Total NCM score	Cognitive appraisal, capability beliefs, and motivational consequences	Internal consistency: Cronbach’s α = 0.80
McDonough et al. (2022) [29]	Medical students and postgraduate trainees	Self-developed questionnaire	2	5-point Likert scale	Agreement with one or both fear-related items (afraid of neurology and neuroscience)	Affective response	Not reported
McGovern et al. (2021) [21]	Medical students	Neuro-Q [21]	5	5-point Likert scale	Neuro-Q score > 16 = neurophobia; Neuro-Q score > 18 = marked neurophobia	Cognitive appraisal and capability beliefs	Internal consistency: Cronbach’s α = 0.58; convergent validity with Schon questionnaire (*r* =.173, *p* =.003);
Medina et al. (2020) [40]	Psychiatry residents	Self-developed questionnaire	1	5-point Likert scale	Self-identification with neurophobia	Affective response	Not reported
Murthy et al. (2023) [10]	Veterinary students	VetNeuroQ	10 (pre-clinical); 14 (clinical)	5-point Likert scale	VetNeuroQ < 12	Cognitive appraisal, capability beliefs, and motivational consequences	Construct validity: CFA with good fit (CFI > 0.9; RMSEA < 0.08) Internal consistency: Cronbach’s alpha > 0.7 across domains
Rodrigues et al. (2023) [33]	Medical students	Neuro-Q [21]	5	5-point Likert scale	Neuro-Q score > 16 = neurophobia; Neuro-Q score > 18 = marked neurophobia	Cognitive appraisal and capability beliefs	Validated instrument (as stated by authors; not detailed)
Saldaña-Inda et al. (2023) [37]	Medical residents	Self-developed questionnaire	1	5-point Likert scale	High or very high fear of neurology and related areas: (Likert 4–5)	Affective response	Not reported
Shiels et al. (2017) [22]	Medical students	Self-developed questionnaire based on Schon et al. [3]	1	5-point Likert scale	Fear of or aversion to neuroscience (Likert 4–5)	Affective response	Not reported

### Quality assessment

First, we ensured a minimum quality standard by including only peer-reviewed journal articles. Subsequently, as a secondary quality assessment measure, we applied the Mixed Methods Appraisal Tool (MMAT [28]. The MMAT contains five checklists corresponding to different study designs (qualitative research, quantitative randomized controlled trials, quantitative non-randomized studies, quantitative descriptive studies, and mixed-methods studies). We selected this tool for its broad scope, as it allowed us to conduct the quality assessment of the diverse study types included in this using a standardized set of criteria. For each study design, we answered five methodological quality questions with “Yes,” “No,” or “Can’t tell,” depending on whether sufficient information was reported in the primary study. Detailed results are presented in Table 2. No study fulfilled all quality indicators. No study was excluded based on the results of the critical appraisal.

**Table 2:** Quality assessment using the Mixed Methods Appraisal Tool criteria

Studies	1. Qualitative studies					2. Quantitative randomized controlled trials					3. Quantitative non-randomized studies					4. Quantitative descriptive studies					5. Mixed methods studies				
	1.1	1.2	1.3	1.4	1.5	2.1	2.2	2.3	2.4	2.5	3.1	3.2	3.3	3.4	3.5	4.1	4.2	4.3	4.4	4.5	5.1	5.2	5.3	5.4	5.5
Aka et al. (2025) [18]											0	1	1	0	0										
Aka et al. (2025) [34]																1	0	1	0	1					
Ali et al. (2025) [35]											1	0	0	0	0										
Ali et al. (2026) [38]											0	1	2	0	1										
Chua et al. (2020) [30]											1	1	1	1	0										
Corigliano et al. (2024) [9]											0	1	1	0	1										
Fantaneau et al. (2014) [2]	1	1	1	1	1						0	0	0	0	0						1	1	1	1	0
Farrag et al. (2025) [36]											1	1	2	0	0										
Han et al. (2023) [32]											1	0	1	0	0										
Holroyd et al. (2025) [19]											1	0	1	0	1										
Jukna et al. (2023) [8]											1	0	1	1	0										
Kam et al. (2013) [39]											0	0	1	1	0										
Lambea-Gil et al. (2023) [31]											0	0	1	0	0										
McElligott et al. (2025) [20]						1	1	0	0	2															
McDonough et al. (2022) [29]											0	1	1	0	0										
McGovern et al. (2021) [21]											2	1	1	0	1										
Medina et al. (2020) [40]											0	0	1	0	0										
Murthy et al. (2023) [10]											0	1	1	1	0										
Rodrigues et al. (2023) [33]											1	1	1	1	0										
Saldaña-Inda et al. (2023) [37]											1	0	1	0	0										
Shiels et al. (2017) [22]											1	0	0	0	1										

Note. 1.1 = appropriateness of approach; 1.2 = adequacy of data collection; 1.3 = adequacy of findings derived from data; 1.4 = interpretation supported by data; 1.5 = coherence across sources, collection, analysis, and interpretation; 2.1 = appropriateness of randomization; 2.2 = comparability of groups at baseline; 2.3 = completeness of outcome data; 2.4 = blinding of outcome assessors; 2.5 = adherence to assigned intervention;3.1 = representativeness of participants; 3.2 = appropriateness of outcome and exposure measurements; 3.3 = completeness of outcome data; 3.4 = confounders accounted for in design and analysis; 3.5 = intervention/exposure administered as intended; 4.1 = relevance of sampling strategy; 4.2 = representativeness of the sample; 4.3 = appropriateness of measurements; 4.4 = low risk of nonresponse bias; 4.5 = appropriateness of statistical analysis; 5.1 = rationale for using a mixed methods design; 5.2 = effective integration of components; 5.3 = interpretation of integrated outputs; 5.4 = divergences between qualitative and quantitative results addressed; 5.5 = components adhere to the quality criteria of their methodological traditions (criteria 1.1–1.5 and 3.1–3.5 assessed in this study as well); 0 = No; 1 = Yes; 2 = Can’t tell

## Results

### General characteristics of the included studies

Regarding the extracted data, Table 3 provides a comprehensive overview of the included studies, detailing sample characteristics, measurement instruments, prevalence estimates, and main findings related to neurophobia.

**Table 3:** Main characteristics and outcomes of articles included in the systematic review

Author (year)	Country		Population	Sample (*n*)	Sex distribution (M/F/NR/NB)	Measurement instrument of neurophobia	Domains assessed	Prevalence (%)	Main findings related to neurophobia
Aka et al. (2025) [18]		Ivory Coast	Medical students	85	54 M/31F	NeuroQ	Cognitive appraisal and capability beliefs	23.35%	No factors analytically associated with neurophobia reported
Aka et al. (2025) [34]		Ivory Coast	Medical students and interns	284	177 M/107F	Two-item difficulty and confidence scale	Cognitive appraisal and capability beliefs	27.46%	No factors analytically associated with neurophobia reported
Ali et al. (2025) [35]		Egypt	Medical students	434	240 M/194F	Two-item difficulty and confidence scale	Cognitive appraisal and capability beliefs	35.7%	No factors analytically associated with neurophobia reported
Ali et al. (2026) [38]		Egypt	Interns/primary care and residents	445	221 M/224F	Two-item difficulty and confidence scale	Cognitive appraisal and capability beliefs	50.4%	No factors analytically associated with neurophobia reported
Chua et al. (2020) [30]	Malaysia		Family medicine specialist trainees	415	115 M/300F	Single-item difficulty rating	Cognitive appraisal	66%	Poor self-rated knowledge, self-declared phobia, perceived importance of basic neuroscience and examination complexity, practicing in the government sector, and perceiving textbooks as not useful independently associated with higher neurophobia
Corigliano et al. (2024) [9]	United States		Dental students	62	21 M/39F/2NR	NeuroQ	Cognitive appraisal and capability beliefs	51.6%	No factors analytically associated with neurophobia reported
Fantaneau et al. (2014) [2]	Canada		Medical students	187	NR	Two-item fear-based scale	Affective response	Fear of neurology: 24%; fear of neuroscience:32%; both: 18%	Qualitative findings (focus groups): non-modifiable factors (prior exposure; preconceptions); modifiable factors including facilitators (clinically based instructors; visual media; neurology texts; case-based learning) and barriers (complex terminology; lack of reinforcement; limited clinical application; didactic methods; feeling lost); changes in perspective (evolving preconceptions; new opinions)
Farrag et al. (2025) [36]	Egypt		Medical students	1235	605 M/630F	NeuroQ	Cognitive appraisal and capability beliefs	26.0% (Helwan); 47.7% (BUC)	Higher NeuroQ scores in male students; higher scores associated with perceived complexity, difficulty applying knowledge, limited clinical exposure, and excessive theoretical content
Han et al. (2023) [32]	China		Medical students and resident trainees	351	146 M/205F	Two-item difficulty and confidence scale	Cognitive appraisal and capability beliefs	66.1% (medical students); 58.6% (resident trainees)	Higher prevalence of neurophobia in medical students compared with resident trainees
Holroyd et al. (2025) [19]	United States		Medical students	362	NR	Two-item fear-based scale	Affective response	NR	No factors analytically associated with neurophobia reported
Jukna et al. (2023) [8]	Lithuania		Medical students	852	191 M/658F/3NB	Two-item difficulty and confidence scale	Cognitive appraisal and capability beliefs	58.9%	Higher neurophobia in exclusively online or face-to-face formats compared with blended learning; positive teaching experience associated with lower neurophobia and greater intention to pursue neurology
Kam et al. (2013) [39]	Singapore		Medical students and junior doctors	289	162 M/127F	Two-item difficulty and confidence scale	Cognitive appraisal and capability beliefs	47.5% (medical students); 36.6% (junior doctors)	Trend towards higher neurophobia in medical students than junior doctors; higher neurophobia in females; neurophobia associated with lower knowledge and interest, non-neurologist teaching, poorer teaching quality, and lack of neurology posting
Lambea-Gil et al. (2023) [31]	Spain		Medical students	320	NR	Single-item fear or aversion scale	Affective response	34.1%	Higher neurophobia in fourth-year students compared with second- and sixth-year students; Students most frequently reported reasons of neurophobia were: limited knowledge in basic neuroscience and predominantly theoretical teaching, with additional factors including poor integration of neuroscience topics, difficulty with neuroanatomy and neurophysiology, limited bedside teaching, and perceived complexity of neurological patients and examination
McElligott et al. (2025) [20]	Ireland		Medical students	77	27 M/50F	NCM	Cognitive appraisal, capability beliefs, and motivational consequences	NR	No factors analytically associated with neurophobia reported
McDonough et al. (2022) [29]	Africa		Medical students and postgraduate trainees	294	148 M/141F/5NR	Two-item fear-based scale	Affective response	26% (neurology); 27% (neuro-science); 32% (≥ 1 domain); 22% (both domains)	Higher neurophobia in women and in those not interested in neurology; qualitative reports described neurology as demanding, associated with poor outcomes, and requiring greater perceived importance
McGovern et al. (2021) [21]	France		Medical students	352	NR	NeuroQ	Cognitive appraisal and capability beliefs	32.4%	Higher NeuroQ scores in female students, mainly in confidence items; higher neurophobia associated with prior perception of neurology as complex; lower scores in students interested in a neurology career
Medina et al. (2020) [40]	United States		Psychiatry residents	183	NR	Single-item self-identification scale	Affective response	30%	Senior residents reported lower self-identification with neurophobia compared with junior residents (*p* =.028)
Murthy et al. (2023) [10]	United States and Canada		Veterinary students	531	68 M/438F/8NB/2NR	VetNeuroQ	Cognitive appraisal, capability beliefs, and motivational consequences	51.6%	Higher VetNeuroQ scores (lower neurophobia) with increasing training exposure, highest after clinical rotations; higher scores also in students with prior neuroscience training or personal connections to neurology
Rodrigues et al. (2023) [33]	Brazil		Medical students	824	323 M/501F	NeuroQ	Cognitive appraisal and capability beliefs	63.3% neurophobia; 28.1% marked neurophobia	Higher neurophobia in fourth-year students; main reported reasons included need to understand neuroanatomy and neurophysiology, complex clinical examination, insufficient teaching time, and perceived difficulty of neurology
Saldaña-Inda et al. (2023) [37]	Spain		Medical residents	134	NR	Single-item fear or rejection scale	Affective response	27.6%	Neurology perceived as highly difficult with low confidence across areas, especially neuromuscular, neuro-ophthalmology, and spinal cord pathology; perceived causes included predominantly theoretical teaching, complexity of patients/diagnoses, and limited patient exposure (more frequent in hospital-based residents)
Shiels et al. (2017) [22]	Grenada		Medical students	446	196 M/204F	Single-item aversion to neuroscience scale	Affective response	19% (first-year); 26% (second-year)	Higher neurophobia in second-year compared with first-year students

Note. *F =* female; *M =* male; *N/A =* not applicable; *NB =* non-binary; *NCM =* Neuro-Combined Measure; *NeuroQ =* Neurophobia Questionnaire; *NR =* not reported; *VetNeuroQ* = Veterinary Neurophobia Questionnaire

The selected articles were published between 2013 and 2026, with most studies published in the last 4 years (*n* = 14; 66.7%), with 2025 being the most productive year, accounting for 28.6% (*n* = 6) of the included studies.

The studies were conducted across 14 countries, including a multicountry sample from Africa [29]. Most studies (90.5%) focused on medical students and physicians in training (including residents, junior doctors, or postgraduate trainees) [2, 8, 18–22, 29–40] followed by studies focused on dental (4.8%) [9] and veterinary students (4.8%) [10]. Sample sizes ranged from 62 to 1,235, although some studies included multiple samples or subgroup analyses. Overall, gender distribution was somewhat unbalanced. Across the 16 studies reporting this information, women accounted for an average of 58.7% of participants. In 9 studies, the distribution was notably skewed (i.e., more than 60% or less than 40% female participants) [8–10, 18, 20, 21, 30, 33, 34]. Five studies did not report gender data [2, 19, 31, 37, 40], and only two included participants who identified as non-binary [8, 10].

### Instruments used to measure neurophobia

Regarding the operationalization of the construct, the instruments used across the selected studies showed substantial heterogeneity in scope, structure, and psychometric rigor. Based on our aforementioned classification (i.e., affective response—fear, aversion; cognitive appraisal—perceived difficulty, complexity; capability beliefs—confidence, self-efficacy; and motivational consequences—interest), we observed distinct measurement patterns across the literature. Specifically, affective response was assessed in 7 studies [2, 19, 22, 29, 31, 37, 40]. In most of them, this domain was measured using one- or two-item Likert scales assessing fear of or aversion to neurology or neuroscience, although in one case, a single item assessing self-identification with neurophobia was used [40]. Notably, in all cases, these measures assessed the affective domain in isolation and were not combined with other domains within the same instrument. Furthermore, no study used a psychometrically validated instrument for this domain.

Conversely, cognitive appraisal and capability beliefs were the most frequently assessed domains and were consistently evaluated together. Cognitive appraisal (i.e., typically operationalized as the perceived difficulty or complexity of neurology) was assessed in isolation in only one study using a single item [30]. In the remaining studies, it was combined with capability beliefs, usually measured as self-reported confidence/ability to understand or apply neurology. This combination was assessed using two types of instruments: two-item Likert-type scales [8, 32, 34, 35, 38, 39] and the multidimensional Neurophobia Questionnaire (NeuroQ) [9, 18, 21, 33, 36]. Regarding the latter, the NeuroQ, the first scale specifically developed to measure neurophobia among medical students, demonstrated limited internal consistency in its original validation (Cronbach’s α = 0.58). Subsequent adaptations have shown similar limitations, with the English adaptations reporting modest Cronbach alpha values [9], while the Portuguese version [33] and other English-language studies have not reported psychometric properties [18, 36].

Finally, motivational consequences were assessed less frequently. Only two studies included measures of interest in neurology, and in both cases, these were assessed within multidimensional instruments that also included cognitive appraisal and capability beliefs [10, 20]. These instruments showed acceptable-to-good internal consistency (Cronbach’s α = 0.80 for the Neuro-Combined Measure (NCM) [20]; α and ω > 0.70 for the VetNeuroQ [10]), although their overall psychometric validation remains limited.

### Prevalence of neurophobia

**Fig. 2:**
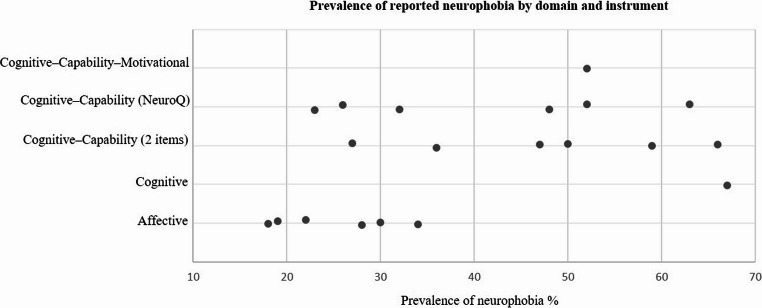
Note. Each point represents one study. Prevalence corresponds to the proportion of participants classified as neurophobic according to each study’s operational definition. Studies are grouped by measurement domain and instrument. Affective refers to measures capturing fear or aversion toward neurology. Cognitive refers to measures assessing perceived difficulty or complexity. Cognitive–Capability (2 items) refers to brief measures combining perceived difficulty and self-confidence. Cognitive–Capability (NeuroQ) refers to studies using the Neurophobia Questionnaire. Cognitive–Capability–Motivational refers to a single study combining perceived difficulty, self-confidence, and motivational components. Horizontal dispersion reflects variability in reported prevalence across studies within each measurement category

In this review, the prevalence of neurophobia, defined as the proportion of participants classified as neurophobic according to each study’s operational definition, was used as a common metric for descriptive comparison. However, given the heterogeneity in instruments and definitions, these results were synthesized narratively. Overall, the reported prevalence of neurophobia showed wide variability across studies, ranging from approximately 19 [22] to 66.1% [32]. Notably, this variability persisted even among studies assessing similar conceptual domains and using comparable measurement approaches (Fig. 2). Specifically, studies measuring the affective response alone (i.e., fear or aversion) showed the lowest variability, with prevalence rates ranging from 19 to 34% [2, 19, 22, 29, 31, 37, 40]. These studies were conducted predominantly with medical students across different stages of training [2, 19, 22, 31, 37], although one study included a mixed sample of medical students and postgraduate trainees [29], and another was conducted exclusively with psychiatry residents [40].

Conversely, studies operationalizing neurophobia through cognitive appraisal and capability beliefs showed substantial variability. Within this category, studies using two-item measures reported prevalence estimates ranging from 27.46 [34] to 66.1% [32], and were conducted primarily with medical students [8, 32, 35], with additional samples including mixed student-intern populations [34], resident trainees [32], and junior doctors [39]. No clear gradient by training stage or participant profile could be identified. Similarly, studies using the NeuroQ reported prevalence between 23.35 [18] and 63.3% [33], and were conducted almost exclusively with medical students [18, 21, 33, 36], with the exception of one study in dental students [9].

Finally, instruments incorporating the cognitive appraisal, capability beliefs, and motivational consequences were used in only two studies. McElligott et al. [20] did not report an explicit prevalence in their sample of medical students, whereas Murthy et al. [10] reported a prevalence of 51.6%. in a sample of veterinary students.

### Factors associated with the development of neurophobia

Regarding intrinsic factors, interest was consistently associated with lower neurophobia in the three studies that examined this variable, despite differences in measurement approaches. Specifically, within the affective domain, McDonough et al. [29] reported that students interested in a career in neurology were less likely to report neurophobia. Conversely, in studies assessing cognitive appraisal and capability beliefs, McGovern et al. [21] found lower NeuroQ scores among students interested in a career in neurology, while Kam et al. [39] identified low interest and low perceived knowledge as independent predictors of neurophobia. Methodologically, these three studies were non-randomized quantitative investigations with sample sizes of approximately 300 participants each and, according to the MMAT criteria, they exhibited low-to-moderate methodological quality. Furthermore, Chua et al. [30] identified significant associations between neurophobia and factors such as self-rated knowledge, self-declared phobia, the perceived importance of basic neuroscience, and examination complexity.

Similarly, gender differences were examined using heterogeneous measurement approaches, primarily within the domains of cognitive appraisal and capability beliefs. Within this group, one study used a two-item measure to capture perceived difficulty and confidence, and it reported higher neurophobia in women [39], while three other studies used the NeuroQ [21, 33, 36]. Among the studies using the NeuroQ, findings were inconsistent: one reported higher levels of neurophobia in men [36], another found no gender differences [33], and a third observed higher neurophobia in women, particularly in confidence-related items [21]. Meanwhile, among the two studies examining gender differences using instruments that assess a single domain of neurophobia, one focused on cognitive appraisal and found no gender differences [30], whereas the other focused on the affective domain and reported higher neurophobia in women [29].

Regarding educational variables, teaching-related factors were examined in a limited number of studies and were classified into specific domains. Within the domains of cognitive appraisal and capability beliefs, clinical teaching delivered by neurologists was associated with lower odds of neurophobia, whereas instruction by non-neurologists was associated with an increased risk [39]. Additionally, teaching methodology was also linked to neurophobia, with students exposed to blended learning reporting lower levels of neurophobia and better academic performance compared to those receiving exclusively face-to-face or fully online instruction [8]. The role of classroom materials and resources has likewise been emphasized. Within the domain of cognitive appraisal, perceiving textbooks as less useful was independently associated with higher neurophobia [30].

Regarding progression in training, findings from affective measures among medical students were derived from two studies that assessed different stages of training. Specifically, Shiels et al. [22] reported higher levels of neurophobia in second-year students compared to first-year students, whereas Lambea-Gil et al. [31] observed a peak in fourth-year students compared to second- and sixth-year students.

Conversely, a study using the NeuroQ, which captures cognitive appraisal and capability beliefs, described a U-shaped pattern, with lower levels in fourth-year students and higher levels in both second-year students and last-year students [33]. Furthermore, in veterinary students, where the VetNeuroQ was used (assessing cognitive appraisal, capability beliefs, and motivational consequences), neurophobia decreased with academic progression, with lower levels observed among those in clinical rotations [10]. Moreover, prior exposure to neuroscience before entering veterinary school was associated with lower levels of neurophobia [10].

**Fig. 3:**
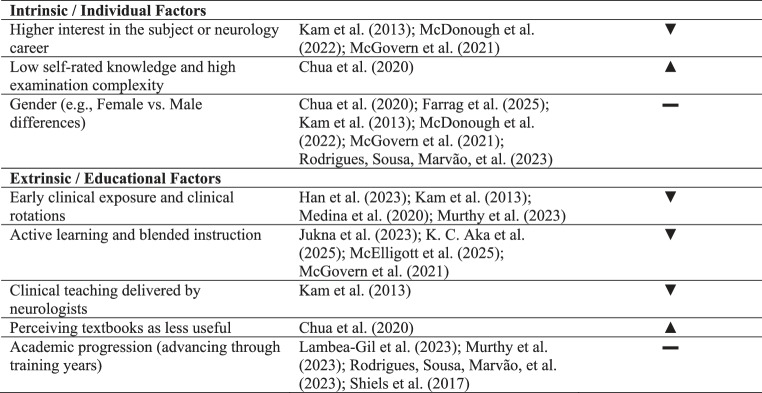
Note. ▼: Associated with a reduction in neurophobia (or lower prevalence). ▲: Associated with an increase in neurophobia (or higher prevalence). ⚊: Inconsistent, mixed (e.g., U-shaped patterns), or no clear association

Finally, clinical experience was consistently associated with lower levels of neurophobia in the studies that examined this factor, all of which were conducted in medical populations, regardless of the domain assessed. Studies examining cognitive appraisal and capability beliefs [32, 39], as well as the affective domain [40], showed that greater clinical exposure was linked to lower neurophobia. Methodologically, all these studies were quantitative, non-randomized, and of low-to-moderate methodological quality. In addition, in veterinary students, additional contextual factors such as having family members or friends working in neurology were also associated with lower neurophobia [10] (Fig. 3).

### Educational interventions

Among the 21 studies reviewed, 6 investigated educational interventions aimed at reducing neurophobia. (Table 4)

**Table 4:** Description of educational interventions, methodologies, and outcomes related to neurophobia

Author (year)	Population	Sample(*n*)	Study design	Type of intervention	Intervention characteristics	Neurophobia measure	Main outcomes related to neurophobia
Aka et al. (2025) [18]	Medical students	Pre: 85 Post: 77	Prospective pre–post study	Bedside clinical training	Supervised bedside neurology rotation in small groups during a compulsory internship (2–3 months)	NeuroQ	Decreased neurophobia; reduction observed in both sexes and in Master’s/Doctorate students, with no change in Bachelor’s students
Corigliano et al. (2024) [9]	Dental students	63 (baseline assessment); 38 (intervention subgroup)	Cross-sectional educational pilot	3D neuroanatomy model-assisted learning	Use of a 3D-printed cerebral arterial circle model during a neuroanatomy lab session alongside cadaveric brains and 2D diagrams	NeuroQ	No intervention effect assessed
Holroyd et al. 2025 [19]	Medical students	362 (263 fifth-year; 99 seventh-year)	Quasi-experimental	Flipped classroom vs. lecture-based teaching	Neurology teaching using a flipped classroom model (pre-recorded lectures and in-class small-group case discussions) versus traditional in-person lectures	Two-item fear-based scale	Decreased neurophobia; reduction limited to fifth-year flipped classroom group; no between-group differences
McElligott et al. 2025 [20]	Medical students	77	Randomized crossover trial	Multimodal educational intervention (ANSWER program)	Analogy-based explanations, clinical integration, embodied simulation, video demonstrations, and retrieval-based learning vs. usual teaching	NCM	Decreased neurophobia; sustained after washout; no change in control group
McGovern et al. (2021) [21]	Medical students	395	Prospective pre–post study	Simulation-based educational program (“The Move”)	Five weekly 3-hour sessions using roleplay to simulate neurological syndromes, with students acting as patients and examiners under neurologist supervision	NeuroQ	Decreased neurophobia; reduction in NeuroQ scores, with a modest reduction in prevalence and greater improvement in students with higher baseline neurophobia
Shiels et al. (2017) [22]	Medical students	150	Prospective longitudinal study	Curriculum-based multimodal teaching (TBL, CBT, PBL)	Integrated neuroscience course including team-based learning, case-based teaching, and problem-based learning sessions throughout the semester	Single-item aversion to neuroscience scale	Increased neurophobia over time; no clear effect attributable to specific interventions

Note. *CBT =* Case-Based Teaching; *PBL=* Problem-Based Learning; *TBL=* Team-Based Learning; *NeuroQ=* Neurophobia Questionnaire; *NCM =* Neuro-Combined Measure

Specifically, we identified two main teaching methodologies across the selected studies: (1) active learning approaches among medical students, including team-based learning, case-based teaching and problem-based learning [22], simulation-based roleplay [21], structured multimodal interventions such as the ANSWER methodology [20], flipped classroom models with in-class interactive activities [19], and hands-on clinical neurology training with bedside teaching [18] and (2) 3D neuroanatomy teaching tools among dental students [9].

Regarding methodological design, five of the six studies that examined the impact of educational interventions on neurophobia compared neurophobia levels before and after the intervention [18–22]. However, the observed effects were highly dependent on the domain assessed, which prevented a direct comparison of results across studies. For instance, the two studies using the NeuroQ, assessing cognitive appraisal and capability beliefs, reported reductions in neurophobia following the intervention [18, 21]. Similarly, the study by McElligott et al. [20], which incorporated cognitive appraisal, capability beliefs, and motivational consequences, also reported reductions in neurophobia. In contrast, studies assessing the affective response showed inconsistent findings: one study reported a partial reduction in neurophobia, limited to a specific subgroup, with no differences between instructional formats [19], whereas another, which was not designed to isolate intervention effects, observed an overall increase following course completion [22]. Corigliano et al. [9], by contrast, measured neurophobia levels only before the intervention, which precluded the examination of the intervention’s effect.

## Discussion

In this review, we systematically examined studies on neurophobia across health-related disciplines, focusing on its prevalence, measurement, associated factors, and educational interventions. For this purpose, we classified the included studies according to the conceptual domains captured by the measurement instruments (i.e., affective response, cognitive appraisal, capability beliefs, and motivational consequences) and synthesized the findings using a structured narrative approach.

Our results indicate that the reported prevalence rates of neurophobia in the included studies showed wide variability, ranging from 19 to 66.1% [22, 32], which is consistent with previous systematic reviews [23–25]. This variability appears to be partly related to the domains assessed. For instance, measures focusing on the affective response yielded lower and more homogeneous estimates [2, 19, 22, 29, 31, 37, 40], whereas those assessing cognitive appraisal and capability beliefs reported higher and more variable prevalence rates [18, 32]. However, the persistence of variability even among studies using the same instrument [18, 21, 33, 36] suggests that contextual factors might also play a substantial role. Differences in medical education systems, including curricular structure, clinical exposure and teaching methods, have been proposed as potential contributors [23, 25, 36].

Regarding intrinsic factors, interest was associated with lower levels of neurophobia across all assessed domains [21, 29, 39], suggesting that this construct is not confined to a single dimension of the student experience. Similarly, Chua et al. [30] linked neurophobia to low self-rated knowledge and high examination complexity. In contrast, findings regarding gender were inconsistent and do not support a clear association. These results suggest a significant conceptual overlap that should be considered when interpreting neurophobia research. On the one hand, measurement instruments operationalize neurophobia through distinct domains, such as affective response (i.e., fear, anxiety), cognitive appraisal (i.e., perceived difficulty), capability beliefs (i.e., self-confidence), and motivational consequences (i.e., interest). On the other hand, some studies analyze variables like interest or perceived difficulty as independent associated factors or predictors of neurophobia. However, these variables are not entirely independent; rather, they may be interrelated and connected to broader constructs such as self-efficacy or domain-specific anxiety. In line with previous educational research [41], lower self-efficacy is typically associated with higher anxiety and an amplified perception of difficulty. Recognizing this overlap helps explain the variability in findings depending on which domains are prioritized by each measurement instrument.

Among extrinsic factors, results related to academic progression were heterogeneous and appeared to depend on both the measurement approach and the population studied. In medical students, affective measures showed an inverted U-shaped pattern, with higher neurophobia in mid-training [31], whereas a Rodrigues et al. [33] using the NeuroQ, found a U-shaped distribution, with higher levels in early and late stages of training. In contrast, among veterinary students, neurophobia decreased with academic progression [10]. These discrepancies might reflect the limited number of studies, differences in measurement domains, and variability in curricular structures across contexts [36]. Furthermore, such patterns may be further influenced by factors such as increasing academic demands in later stages [42] or higher anxiety levels in earlier phases of training [43].

By contrast, the relationship between clinical exposure and neurophobia was more consistent. Across studies and domains, greater clinical experience was associated with lower levels of neurophobia [10, 32, 39, 40], suggesting that this effect may extend across different domains of the construct, which is congruent with broader evidence indicating that early clinical exposure in health science students reduces anxiety, enhances self-efficacy, and facilitates the integration of theoretical and practical knowledge [36, 44]. Interestingly, even indirect exposure, such as having family members or friends working in neurology, was linked to lower neurophobia [10]. This finding underscores the potential importance of familiarity and context in the learning process.

Teaching-related variables were also associated with neurophobia, particularly within the domains of cognitive appraisal and capability beliefs. Specifically, clinical teaching delivered by neurologists [39] and the use of blended learning approaches were linked to lower levels of neurophobia [8], whereas perceiving textbooks as less useful was associated with higher levels [30]. Although based on a limited number of studies and self-reported measures, these findings suggest that teaching-related factors may play a relevant role in shaping neurophobia. Also, regarding the efficacy of specific strategies, the available evidence on educational interventions is limited and methodologically heterogeneous. Although most studies included in this systematic review employed active learning approaches in medical education [18–22], the interventions differ substantially in their design, duration, and pedagogical components. We observed that interventions incorporating clinical exposure or applied learning strategies tend to report reductions in neurophobia, particularly in the cognitive and capability domains [18, 20, 21]. Conversely, findings based on affective measures are less consistent, with limited or context-dependent effects [19].

Taken together, these results suggest that neurophobia may be less of an intrinsic difficulty of neurology and more a consequence of how it is taught. The finding that greater clinical exposure is associated with lower neurophobia [10, 32, 39, 40], together with reductions reported following educational interventions incorporating clinical or applied components [18, 20, 21], supports the view that educational experiences may play an important role in its development. In this context, recent studies in neurology education have highlighted structural limitations in training that extend beyond the undergraduate level. Variability across residency programs, along with differences in access to clinical environments and opportunities for skills development, have been identified as key issues [13]. In parallel, deficits in training and unmet needs in specific clinical areas have been described, combined with a demand for more practical and applied learning [45]. These findings reinforce the relevance of clinical exposure, supervised practice, and the integration of theoretical and clinical knowledge as central components of effective neurology or neuroscience training.

This review extends previous systematic work on neurophobia. Previous reviews have focused primarily on medical students and physicians [23–25], whereas the present synthesis broadens its scope to include other health-related disciplines, such as dentistry and veterinary medicine, in which neurophobia has also been documented. Unlike prior work, which did not systematically examine how neurophobia is operationalized [23–25], our study classifies measurement instruments according to their underlying conceptual domains, revealing substantial heterogeneity and limited psychometric validation. In addition, although some reviews have considered educational interventions [24], they have not systematically synthesized them in relation to specific neurophobia outcomes. In this respect, the present review incorporates and examines the available evidence on interventions that explicitly measure this construct. Taken together, these differences position this review as a broader and more structured synthesis of how neurophobia is conceptualized, measured, and addressed across health education and professional training.

### **Strengths and limitations**

The strengths of this review lie in its broad scope across health disciplines, the explicit focus on measurement tools, their classification according to underlying conceptual domains, and the synthesis of evidence on educational interventions. However, significant limitations remain. The studies included were heterogeneous in design, sample characteristics, and instruments employed, which hinders comparisons across contexts. The limited availability of standardized and validated measures of neurophobia is a major barrier, and most research has been conducted in medical populations, with very few studies exploring other health-related disciplines. Publication bias and language restrictions may also have influenced the findings.

### **Future directions**

Advancing research on neurophobia requires the development and adoption of validated instruments that adequately capture its multidimensional nature, enabling more accurate assessment of its prevalence, determinants, the effectiveness of educational interventions, and comparability across studies. Future studies should also examine neurophobia across its underlying domains to determine whether different dimensions respond differentially to educational strategies. In addition, future research should systematically examine the role of individual neurophobia factors, such as self-efficacy and anxiety, as these remain largely unexplored despite their established relevance in educational psychology. Educational interventions should be evaluated through controlled experimental designs and across different health disciplines to determine their effectiveness and generalizability.

## Conclusions

Neurophobia emerges as a multidimensional construct that extends beyond affective responses to include cognitive appraisal, capability beliefs, and motivation. Available evidence suggests that its prevalence varies across measurement instruments and may be influenced by educational and contextual factors. In particular, associations with clinical exposure and the results of educational interventions suggest that neurophobia might be more closely related to difficulties in translating theoretical knowledge of neurology and neuroscience into clinical practice than to an intrinsic complexity of these disciplines. However, the current evidence is limited by heterogeneity in study design and measurement instruments, as well as by the limited availability of adequately validated tools. Addressing these limitations and strengthening the integration between theoretical and clinical training is key to better understanding and reducing neurophobia across health disciplines.

## Data Availability

All data supporting the findings of this study are available within the cited articles and the tables presented in this manuscript. No additional datasets were generated or analysed.

## Declarations

### AI assistance statement

Generative AI (ChatGPT, OpenAI, version GPT-5.1) was used solely to assist with English language editing and refinement of phrasing during the preparation of this manuscript. The tool did not generate research content, conduct analyses, extract data, or contribute to the interpretation of findings. In addition, the manuscript underwent professional human language revision by a qualified translator. All substantive ideas, analytical decisions, and final revisions were made entirely by the authors.
